# Biomarkers in Thyroid Cancer: Emerging Opportunities from Non-Coding RNAs and Mitochondrial Space

**DOI:** 10.3390/ijms25126719

**Published:** 2024-06-18

**Authors:** Patricio Cabané, Claudio Correa, Ignacio Bode, Rodrigo Aguilar, Alvaro A. Elorza

**Affiliations:** 1 Department of Head and Neck Surgery, Clinica INDISA, Santiago 7520440, Chile; patricio.cabane@indisa.cl (P.C.); c.correallanos@uandresbello.edu (C.C.); 2 Faculty of Medicine, Universidad Andres Bello, Santiago 8370071, Chile; 3 Department of Basic and Clinical Oncology, Faculty of Medicine, University of Chile, Santiago 8380453, Chile; 4 Institute of Biomedical Sciences, Faculty of Medicine and Faculty of Life Sciences, Universidad Andres Bello, Santiago 8370071, Chile; ignacio.bode@gmail.com

**Keywords:** thyroid cancer, diagnosis, molecular markers, non-coding RNAs, mitochondria

## Abstract

Thyroid cancer diagnosis primarily relies on imaging techniques and cytological analyses. In cases where the diagnosis is uncertain, the quantification of molecular markers has been incorporated after cytological examination. This approach helps physicians to make surgical decisions, estimate cancer aggressiveness, and monitor the response to treatments. Despite the availability of commercial molecular tests, their widespread use has been hindered in our experience due to cost constraints and variability between them. Thus, numerous groups are currently evaluating new molecular markers that ultimately will lead to improved diagnostic certainty, as well as better classification of prognosis and recurrence. In this review, we start reviewing the current preoperative testing methodologies, followed by a comprehensive review of emerging molecular markers. We focus on micro RNAs, long non-coding RNAs, and mitochondrial (mt) signatures, including mtDNA genes and circulating cell-free mtDNA. We envision that a robust set of molecular markers will complement the national and international clinical guides for proper assessment of the disease.

## 1. Introduction

Thyroid cancer accounts for 1 to 4% of all malignancies worldwide. Its incidence has increased over the years, from 1.5% before 1996 to 6.8% after 1996, with an increase of three times per decade [1] (for a definition of epidemiological terms, see Table 1). In Chile, the estimated number of new cases per year is 1300 [2]. This increase in incidence has been attributed to a higher diagnosis rate, mainly due to the implementation of imaging techniques (diagnosis achieved in 50% of cases), as well as genetic and environmental factors, such as obesity and the increase in iodine consumption [1]. Histologically, thyroid cancer develops in two cell types, with more than 90% derived from follicular cells (epithelial, responsible for the uptake of iodine and production of thyroid hormones), and 3–5% derived from parafollicular cells (associated with synthesis and production of calcitonin).

According to the WHO 2022 guidelines, thyroid cancers have been categorized into three primary groups based on their level of differentiation. (1) Low grade (differentiated) cancers, which include papillary thyroid carcinoma (PTC), follicular thyroid carcinoma (FTC) and oncocytic carcinoma. The prognosis for these cancers is generally favorable. (2) High-grade cancers, which include poorly differentiated thyroid carcinoma (PDTC) and high-grade differentiated thyroid carcinoma (DHGTC). These cancers are more aggressive, presenting poorer outcomes, than low-grade cancers. (3) Anaplastic thyroid cancer (ATC), which is the most undifferentiated and aggressive form of thyroid cancer with a very poor prognosis [3]. Papillary thyroid cancer is the most common and has several histological subtypes (diffuse sclerosing, tall cell, columnar cell, solid, and hobnail among others) and clinical behavior [4]. Although we acknowledge different tumor types of thyroid carcinomas, we mostly focus on the papillary thyroid carcinoma in this review.

### 1.1. Diagnosis and Uncertainty in Thyroid Cancer 

The classic methods for diagnosing thyroid cancer are through images (ultrasound—TIRADS classification) and fine-needle aspiration (cytology) [5,6]. The latter allows classifying the probability of thyroid cancer according to the “Bethesda Criteria” from I to VI: I, non-diagnosed or inadequate; II, benign; III, atypia of undetermined significance; IV, follicular neoplasia; V, suspicious for malignancy; and VI, malignant. In the latest update (2023), each category receives a combined risk of malignancy (ROM), with a mean of 13%, 4%, 22%, 30%, 74%, and 97%, respectively [6]. Handling cases based on an indeterminate cytological study is not solely determined by the Bethesda classification. In fact, the application of this method alone has not been able to provide clinically applicable pre-surgical recommendations, either in terms of follow-up or diagnostic surgical resection. As a result, international guidelines incorporate clinical, ultrasound, and cytological characteristics, as well as molecular markers [7]. The latter tests are based on genetic mutation analysis, gene expression evaluation, and microRNA-based classifiers (specific panels are not disclosed). Genetic mutations related to the mitogen-activated protein kinase/extracellular signal-regulated kinase (MAPK/ERK) pathway are at the forefront of research in this area [8]. Clinical and molecular tools for guiding the diagnosis and proposing therapeutic options for thyroid cancer patients based on molecular characterization are limited and have not been updated so far. Some new tumorigenic and prognostic factors have been added to the study of other tumors, and more recently, to thyroid cancer [5,9,10]. The application of these markers in clinical decision-making should be studied in detail. This review provides a comprehensive overview of potential new markers with higher clinical relevance in thyroid cancer.

### 1.2. Current Preoperative Molecular Markers and Commercial Tests

There are molecular tests and tumor markers to study thyroid cancer, particularly the papillary thyroid carcinoma, but there is also an availability of molecular tests to study the other histological types of thyroid neoplasia [7,11,12]. Molecular genetic markers, such as mutations in MAPK pathways, including *ALK*, *NTRK*, *RET*, *RAS*, and BRAF^V600E^, have been studied for the early detection of papillary cancer [13]. Mutations in *RAS*, BRAF^K601E^, *PAX8*/*PPAR* and less common *PTEN* have been observed in follicular thyroid cancer [14,15,16]. For less differentiated tumors with a worse prognosis, such as anaplastic thyroid cancer, where *TERT* and *P53* mutations are common, the integration of these markers will provide better diagnostic and prognostic value.

The indication to perform a molecular study arises in order to safely assign a benign status for an indeterminate thyroid nodule, i.e., reported as Bethesda III or IV [17,18], leading to the avoidance of unnecessary surgical procedure. Molecular studies also allow prognostic stratification and thus modify management decisions, although in our experience routine practice is difficult to massify due to costs and variability between tests [5]. Testing for single mutations, such as BRAF^V600E^ or *RET*/*PTC* translocations, has yielded good specificity (100%) but poor sensitivity (50–60%) [19,20], hence panels detecting gene mutation profiles or changes in expression levels have arisen. It has been proposed that, while mutation tests are good rule-in tests for thyroid cancer, gene expression classifier testing is a good rule-out test for thyroid cancer [13]. The use of *gene mutation profiling panels*, such as a panel detecting mutations found in 70% of thyroid cancers (BRAF^V600E^, *HRAS* codon 61, *KRAS* codons 12/13, *NRAS* codon 61, *RET*/*PTC1*, *RET*/*PTC3*, and *PAX8*/*PPARG*, [9]), has yielded a sensitivity of >90% [21]. A gene classifier test, capable of classifying changes in the expression levels of up to 167 genes, has a significant negative predictive value but little positive predictive value (52%) [9]. 

Regarding commercially available molecular tests used in clinical practice, Table 2 summarizes the main features of Afirma^®^, Thyroseq^®^, Thyroid Print, and ThyGeNEXT^®^, all belonging to a group that identifies genetic anomalies through sequencing and gene expression. 

While Afirma GSC is a rule-out test focused on Bethesda III/IV samples, Afirma XA (Xpression Atlas) is used for GSC suspicious and Bethesda V/VI samples [23]. Studies have shown that complementation of the GEC (Gene Expression Classifier) with the Afirma GSC approach leads to higher specificity without compromising sensitivity [31]. GSC can also be used on its own for benign calls [32,33], and XA improves stratification [34]. When used in combination, GSC and XC presented a high positive and negative predictive value and decreased the number of surgeries in 75% of instances [22]. Thyroseq V3 (2018) is a test based on 112 thyroid-related genes, looking for point mutations, indels, gene fusions, copy number alterations, and abnormal gene expression [25]. In return, Thyroseq reports a “genomic classifier” to separate malignant from benign lesions, preventing surgery in most patients [24]. Regarding Thyroid Print, its recent validation from two multicenter and independent clinical trials has given it a specificity of 88%, a negative predictive value of 95%, and a positive predictive value of 78%. Meanwhile, a study reported a positive predictive value of 71% despite a smaller sample [30]. In Chile, Olmos et al. [29] carried out a prospective cohort study using 1272 fine-needle aspirations in a single-health center. They studied indeterminate nodules (Bethesda III and IV) using the Thyroid Print classifier and found benign nodules (comparable to Bethesda II) in 67% of cases after a 15-month follow-up. For patients with suspicious results after the test, the risk of malignancy was estimated at 71% compared to the histological result, making the risk of malignancy comparable to Bethesda [29]. Finally, the ThyGeNEXT test looks for mutations in selected genes (*ALK*, *BRAF*, *GNAS*, *HRAS*, *KRAS*, *NRAS*, *PIK3CA*, *PTEN*, *RET*, and *TERT* promoter genes) plus fusion genes (*ALK*, *BRAF*, *NTRK*, *PPARG*, *RET*, *PAX8*, *TBP*, *USP33*, and *THADA)* [27], although this panel is often used in combination with a panel of microRNAs (miRNAs) named ThyraMIR to improve stratification and predictive value (more below) [26]. 

The current molecular classifiers have theoretically higher performance in thyroid cancer diagnosis than cytology, with sensitivities ranging from 91–94% and specificities between 68% and 90%. These molecular classifiers have been shown to provide favorable outcomes in cytologically indeterminate nodules, primarily by averting diagnostic surgeries. They achieve this through high negative predictive values, indicating a low likelihood of cancer when test results are negative. However, their widespread application is hindered by the limitation of larger studies outside the United States, either due to the high acquisition costs or logistical terms of commercialization due to patents from the country of origin (USA) and other factors that could be the real causes of exemption from diagnostic surgery in their reports. Despite the use of biological markers in indeterminate nodules following Bethesda classification, there is still no genetic characterization that works independently to select a specific treatment. However, both European and American guidelines suggest interpretation at the clinician’s discretion to guide management if they are available for use [5].

The large genetic panels available on the market, as well as the study of specific mutations (which may or may not be included in the panels), have not only complementary applications with cytology but also in determining the degree of aggressiveness and response to treatment. The mutations BRAF^V600E^, *TERT*, and *RAS* are the most widely studied in relation to these clinical variables [35,36]. Genetic mutations have been observed in various types of thyroid cancer, which have affected the different clinical pillars related to this condition. The presence of BRAF^V600E^ is associated with the classic papillary type and its high cell subtype [35]. It is often linked to locoregional invasion, lymph node metastasis (especially cervical and lung), and refractoriness to radiotherapy. Mutations in *RAS* and *RET* are both related to invasive behavior (*RAS* mostly through the hematogenous route and consequently to the bone) but with differences [37]. Mutations in *RAS* can be present in different types of thyroid cancers (papillary and follicular), with the papillary type having the highest frequency at 38%. These mutations retain susceptibility to radioactive iodine therapy. Additionally, in follicular cell cancers, *RET*/*PTC* fusions (particularly *RET*/*PTC1* and *RET*/*PTC3*) have been identified, especially in the papillary type in pediatric and young adult populations. Their presence indicates a certain behavior but still responds well to radioiodine treatment [35,38]. On the other hand, point mutations in *RET* (specific base pair changes) have lower susceptibility to remission with I131 and are found in medullary thyroid cancer, mainly in hereditary cases, and to a lesser extent in sporadic cases [35].

Based on various histopathological findings in large patient cohorts, the use of molecular markers has been incorporated specifically after cytological diagnosis to determine aggressiveness and response to treatment. However, this is limited to anaplastic and medullary cancer. In the context of anaplastic thyroid cancer, the identification of the BRAF mutation V600E is incorporated into the initial management process to recommend the use of selective BRAF/MEK inhibitors (dabrafenib/trametinib, respectively), particularly if the cancer is not entirely unresectable [35]. When it comes to medullary cancer, detecting the germline mutation in the *RET* gene is used to determine whether it is hereditary [39].

### 1.3. Stratifying the Risk of Disease Recurrence

The standard treatment for differentiated thyroid carcinoma (DTC) usually involves surgery, followed by radioiodine (RI) ablation if necessary. The American Thyroid Association (ATA) suggests classifying patients into three risk groups (high, intermediate, and low) based on the risk of disease recurrence or persistence [17]. In low-risk patients, radioiodine therapy is generally not recommended as a complementary treatment. These patients have a low mortality rate, with a recurrence or persistence risk of about 3% [40]. The ATA guidelines consider the *BRAF* gene mutation as one of the factors to predict intermediate risk of recurrence if it is associated with multifocal micropapillary thyroid cancer. Other risk factors are determined exclusively from the histopathological results following surgery. However, the *BRAF* gene mutation alone cannot determine the use of I131 as it depends on the histological characterization [35]. However, studies have shown that the presence of BRAF downregulates iodine–sodium transporters, regardless of whether it is a classic subtype of papillary thyroid cancer or a cell type, making them resistant to iodine treatment. Therefore, the molecular characterization of thyroid cancer is improved by attempting radiotherapy and, if refractory, scaling up to selective NTRK inhibitors. This reflects the usefulness of incorporating biomarkers into stratification plans, contrasting with the selective use offered by ATA [41,42]. It is worth noting that the markers we described earlier are not the only ones available. New tumor factors have been incorporated into various studies, with and without applications in thyroid cancer. 

## 2. Emerging Molecular Markers for Thyroid Cancer: Opportunities from the Non-Coding Genome

The sequencing of the human genome revealed that most RNAs in eukaryotes are “non-coding” (ncRNA), as they do not have the potential to produce proteins [43]. Thus, 75% of the information contained in DNA is transcribed to RNAs, but only 2% is translated to proteins [44]. Originally, non-coding RNAs were interpreted as transcriptional noise [45]; however, the significant role played by these RNAs has been confirmed, both in biological mechanisms [46,47] and in the development of diseases, including cancer [48,49]. With the advancements and reduced costs in omics technologies, alongside the development of lab-on-chip devices, detecting changes in gene expression or mutations is becoming increasingly accessible and affordable [50,51].

### 2.1. Micro RNAs

MicroRNAs (miRNAs) are non-coding RNAs that are synthesized from longer transcripts and processed into 19- to 25-nucleotide oligonucleotides through a step-wise mechanism (reviewed in [52]). By binding to target mRNAs, miRNAs repress translation and induce mRNA decay, significantly influencing the overall gene expression profile in cells. In thyroid cancer, miRNA expression profiles analyzed through quantitative PCR have been employed to stratify tumors, with diagnostic tests such as ThyraMIR and mir-THYpe being notable examples.

As mentioned before, ThyraMIR is often used as a complement to the ThyGeNEXT test [26]. ThyraMIR analyzes changes in the expression of 11 miRNAs and their association with malignancy (Table 3) [53]. In a single-health center study, ThyGeNEXT + ThyraMIR showed a 52,94% of positive predictive value [22], and 95% of diagnostic yield [54]. Although testing positive is associated with malignancy in surgical pathology, the ThyraMIR classifier failed to differentiate between benign and malignant RAS-mutated tumors [55]. As for mir-THYpe, it analyzes 11 miRNAs, where 6 are used as normalizers (let-7a, miR-103, miR-125a-5p, let-7b, miR-145, and *RNU48*) and 5 as discriminators of malignancy (Table 3) [56]. mir-THYpe reached average values of sensitivity 89.3%, specificity 81.6%, NPV 95%, and PPV 66%, as shown by a prospective, non-interventional multicenter study carried out on 440 patients. These results allowed clinicians to reduce unnecessary surgery in 52.5% of cases [57]. 

It is envisioned that an increased array of molecular biomarkers will enhance the reliability of thyroid cancer tests and offer clinicians more options for stratifying indeterminate thyroid nodules. With the growing number of studies investigating the molecular mechanisms underlying thyroid cancer, additional miRNAs are emerging as potential alternatives for future diagnostic tests [59]. Interestingly, DICER, the endoribonuclease responsible for generating mature miRNAs from stem-loop miRNAs [52], may harbor pathogenic somatic mutations that alter miRNA-5p to miRNA-3p production in thyroid cancer [62]. 

Potential miRNA candidates for diagnosis of thyroid cancer are described in Table 4. Based on the number of studies performed with each miRNA in cancer cells, the miR-1, miR-2, miR-7, and miR144 arise as the most robust and promising biomarkers to be included in future diagnostic tests. With the advancement and refinement of small RNA purification techniques, coupled with the availability of commercial adapters, quantification of miRNAs via real-time PCR or next-generation sequencing platforms has become a routine procedure [50].

### 2.2. Long Non-Coding RNAs (lncRNAs)

Long non-coding RNAs (lncRNAs) are transcripts longer than 200 nucleotides that do not encode proteins. These RNAs are often spliced, capped, and polyadenylated [47]. lncRNAs can modulate gene expression through various mechanisms, including direct binding to the DNA, interacting with epigenetic modifiers, and recruiting transcription factors to regulatory regions. Additionally, lncRNAs regulate miRNA function by capturing them and preventing them from targeting mRNAs in cells. This mechanism prevents miRNAs from blocking translation. Consequently, when lncRNA are dysregulated in disease, associated changes in gene expression may occur at both the transcriptional and post-transcriptional level [84].

Most lncRNAs exhibit cell-specific expression patterns and are typically expressed at low levels [85]. Alterations in their expression have been linked to the development and progression of various diseases, including thyroid cancer [86]. While lncRNAs have been studied in the context of thyroid cancer, their clinical application remains in the research phase. Using long non-coding RNAs (lncRNAs) as biomarkers offers several advantages over proteins, particularly in terms of quantification. Designing, acquiring, and standardizing primers for real-time PCR is often simpler and more straightforward than developing antibodies. Additionally, while messenger RNA levels do not always correlate with protein amounts due to post-transcriptional mechanisms, non-coding RNAs are generally considered more consistent as final products. Although lncRNAs can undergo modifications, such as adenosine methylation, the implications of these modifications are still being explored.

Table 5 presents potential lncRNA candidates for use as biomarkers in thyroid cancer. While all of them may be deemed promising molecules, *HOTAIR* and *MALAT1* stand out as robust candidates due to the substantial support from numerous studies across various cancers [87], along with *PTCSC3* as an RNA originally discovered in thyroid cancer [88]. *HOTAIR* is a lncRNA with an essential role in the repression of early development-related genes in adult individuals, collaborating with epigenetic complexes [89]. In papillary thyroid cancer, *HOTAIR* has been found to be upregulated, leading to increased growth, migration, and invasive properties [76,79,90]. Patients with lower levels of *HOTAIR* have better survival rates [91]. Regarding *MALAT1*, this lncRNA is widely recognized among cancer researchers. Initially discovered as one of the most overexpressed RNAs in lung cancer patients with metastasis, *MALAT1* has been found overexpressed in numerous other tumor types, including thyroid [87,90]. *MALAT11* regulates expression of target genes by directly binding to the genome and collaborating with epigenetic complexes. By functioning also as a competing endogenous RNA, it captures miRNAs in the nucleus [92,93]. Thus, *MALAT1* increases the expression of proteins associated with proliferation, migration, and angiogenesis, such as FGF2. This association links *MALAT1* to a prognosis of metastasis and early locoregional invasion [94]. In anaplastic thyroid carcinoma, *MALAT1* knockdown leads to reduced cell proliferation, invasion, and migration [95], like the effects observed with BRAF and RET gene alterations. This phenomenon has been particularly noted in medullary thyroid cancer [94]. However, its application in clinical practice is currently limited, as it has only been tested in vitro in thyroid tissue [95]. Finally, regarding *PTCSC3*, this lncRNA was initially identified as thyroid-specific [88], although subsequent research has revealed its presence in other cancers [96]. In thyroid cancer cells, it is frequently downregulated [88], and its overexpression has been associated with growth inhibition, cell cycle arrest, and increased apoptosis [97]

### 2.3. Emerging RNA Molecules: Circular and Double-Stranded RNAs

In recent years, RNA variants that were originally disregarded as mis-splicing artifacts or by-products have been found to play significant roles in biological models. Advances in RNA-sequencing techniques and bioinformatics have revealed that circular and double-stranded RNAs participate in various biological mechanisms [107,108]. Circular RNAs are generated by back-splicing of pre-mRNAs and can function by sponging miRNAs and interacting with proteins (see [108] for review). By designing primers flanking the back-splice site, circular RNAs can be quantified specifically. Examples of circular RNAs found to be dysregulated in thyroid cancer cells include circITGA7, circRAPGEF5, circ_0058129, and circ_0005273 [74,81,83]. However, further research is required to gather robust data and propose circular RNAs as clinical biomarkers.

Double-stranded RNAs (dsRNAs), initially associated with viral infections, accumulate in vertebrate cells during the dysregulation of various cellular processes, including cancer [108] These dsRNAs originate from activated long and short interspersed elements, endogenous retroviruses, and mitochondrial transcripts. Unlike dsRNAs derived from viruses, endogenous dsRNAs often evade degradation and remain localized within the nucleus and mitochondria. Their quantification requires antibodies or dsRNA-binding proteins [109], making their incorporation into routine clinical assays more challenging compared to miRNAs or lncRNAs. Currently, no promising candidates among double-stranded RNAs have been identified as potential biomarkers for thyroid cancer diagnosis. Further research will uncover robust biomarkers that could aid in diagnosing cancer patients in the future.

## 3. Opportunities beyond the Nucleus: Mitochondrial Signatures

The proliferative and invasive capacity of cancer cells involves a reprogramming of the intermediary and energy metabolism of tumor cells, where mitochondria are the key players. Beyond energy generation, mitochondria are considered a powerful signaling platform for tumor anabolism and have an impact on tumor redox environment, calcium homeostasis, transcriptional and epigenetic regulation, and cell death pathways. In fact, several anti-cancer treatments disrupt mitochondrial function to trigger cancer cell death [110]. The proper work of mitochondria is sustained by two fundamental processes: first, the oxidative phosphorylation system (OXPHOS), which is the engine of energy generation in the form of ATP from the oxidation of glucose and fatty acid molecules. When mitochondria generate ATP, reactive oxygen species (ROS) are also formed as a by-product which are responsible for many of the aging-associated diseases, including cancer [111]. Second is the mitochondrial life cycle. This cycle refers to mitochondrial dynamics (MtDy), i.e., fusion and fission events of mitochondria; mitophagy, which corresponds to the selective degradation of damaged mitochondria or those that are no longer needed; and Mitochondrial Biogenesis, which involves mtDNA replication, protein synthesis and membrane biosynthesis [112].

### 3.1. mtDNA Signatures

Mitochondria contain multiple copies of mtDNA, which can range from tens to hundreds depending on the physiological and pathophysiological conditions of cells. The mtDNA is highly polymorphic and maternally inherited, which configures everyone with its own molecular maternal surname, called haplogroups, which correlate with the geographic origins of populations. The oldest haplogroups are from Africa and, with geographic migration and climate adaptations, the haplogroups from Europe, Asia, and America have evolved [113]. The type of haplogroup grants adaptive advantages and/or susceptibility to disease [114]. The mtDNA is a 16 kb circular DNA and encodes for 37 genes (13 mRNA, 2 rRNA, and 22 tRNA). The mitochondrial mRNAs encode for 13 core proteins of OXPHOS, which play a critical role in the proper assembly and functioning of the electron transport chain (ETC) for the generation of the mitochondrial membrane potential and, therefore, for the ATP synthesis. As a by-product of ETC functioning, ROS are generated. Since mtDNA molecules are in the same place in which ROS are produced, that is in the mitochondrial matrix, they are prone to undergo point mutations, deletions, and a reduction in their copy number. The mutation rate of mtDNA is higher compared to the nuclear genome [115] and these mutations accumulate as organisms age. The co-existence of wild type mtDNA with mutant mtDNA is known as heteroplasmy [116,117]. mtDNA alterations are associated with severe mitochondrial dysfunction and diseases. Thus, the integrity of mtDNA is pivotal for cell survival and health of the organism [118,119].

Recycling of oxidized and damaged mitochondria, (known as mitophagy) is critical for maintaining the copy number and integrity of mtDNA while keeping reduced levels of heteroplasmy [120,121,122]. Elevated mtDNA heteroplasmy leads to increased ROS which damages the bioenergetic capacity of mitochondria and alters mitophagy, favoring the development of diseases such as cancer [123,124]. Thus, both mtDNA and mitophagy are interconnected and dependent on each other. When mitochondria are stressed and/or mitophagy is dysfunctional, mtDNA may leak out from mitochondria and cells to reach plasma circulation, where it can be detected. The amount and features of this circulating cell-free mtDNA (ccf-mtDNA) constitute a parameter of mitochondrial dysfunction and disease.

In the context of a disease, the detection and analysis of mtDNA in terms of copy number, heteroplasmy, and haplogroups, as well as the levels of ccf-mtDNA, which are all measurable parameters, may configure a genomic signature and a powerful diagnostic tool in cancer. In fact, mtDNA copy number alterations contribute to the development of different types of carcinomas, such as lung cancer [124], breast cancer [125], hepatic cell carcinomas [126], gastric cancers [127], bladder, esophageal, kidney, and head/neck squamous carcinomas [125]. An increased mtDNA copy number may promote cancer progression by enhancing mitochondrial oxidative phosphorylation, as has also been observed in colorectal cancer [128]. Thyroid tumor cells also present an increase in the mtDNA copy number [129]. However, the relationship of mtDNA copy number with the risk of cancer is still inconclusive.

In some cancer cells, the striking accumulation of mitochondria leads to swollen cells with distinctive granular cytoplasmic eosinophilia forming an “oncocytic tumor” [130]. These tumors present two characteristic mutations: a deletion of 4977 bp called “common deletion” [131] and Complex 1 mtDNA mutations (loss of function and missense) that affect nearly 60% of patients [132,133,134]. Regarding papillary thyroid cancer, it was recently reported that this type of tumor also presents mitochondrial accumulation, DNA mutations, and loss of complex I integrity [135]. As for the mitochondrial accumulation, the Tall Cell subtype of Papillary Cell Carcinoma is considered “mitochondria-rich”, indicating that there is a spectrum in mitochondria content that can be indicative of disease progression [135]. In thyroid cancer, an increase has also been reported in the mtDNA heteroplasmy at the level of both point mutations and deletions, mainly affecting the respiratory complex I and IV [136]. Sasarman et al. [137] also reported mutations in *MT-CYB* encoding for Cyt b (subunit of complex III) and *MT-TL1* encoding the mitochondrial tRNA^Leu^ in cancer. In addition, mtDNA mutations affecting the complex I subunit, ND1 (G3842A), ND4 (A11708G), and ND5(12418insA), have been correlated with liver cancer progression, and the ND2 (T4216C) mutation with colorectal cancer [138,139,140]. 

On the other hand, the haplogroup M has been associated with breast cancer [25] while, in thyroid cancer, a preponderance has been observed a preponderance of the haplogroup D4a in Chinese patients [141]. and notably, the haplogroup K in Europeans has been shown to be protective against thyroid cancer [142]. Su et al. [143] reported three sub-haplogroups of N (A4, B4a and B4g) and eight single-nucleotide polymorphisms (mtSNPs) (A16164G, C16266T, G5460A, T6680C, G9123A, A14587G, T16362C, and G709A) associate with the occurrence of papillary thyroid cancer.

Regarding the ccf-mtDNA, accumulating evidence suggests that plasma or serum ccf-mtDNA levels are significantly different between cancer patients and healthy individuals. Thus, the level of ccf-mtDNA has been shown to be significantly higher in patients with lung cancer than in healthy patients [144]. In patients infected with hepatitis B virus (HBV), a lower content of ccf-mtDNA had a significantly increased risk of hepatocellular carcinoma as compared with HBV patients with higher ccf-mtDNA [145]. In metastatic colorectal cancer, a decrease in ccf-mtDNA has also been shown, as compared with healthy individuals [146]. Similar results were found in patients with papillary thyroid carcinoma, who presented decreased ccf-mtDNA levels in plasma [147]. Interestingly, the amount of ccf-DNA in blood has been used as a potential non-invasive biomarker for diagnostic and prognostic evaluation in a wide variety of cancers, because it has been shown to be increased or decreased in the serum of patients with various malignant tumors as compared with healthy patients [148,149]. 

ccf-mtDNA is widely recognized as a favorable option over nuclear-circulating DNA because it is more abundant and then easy to detect and analyze, and because changes in ccf-mtDNA are closely related to the occurrence and development of tumors in terms of copy number and integrity [150]. Thus, the detection and analysis of ccf-mtDNA may provide important information for diagnosis and prognosis in thyroid cancer patients, which may reduce the number of unnecessary thyroid surgical procedures. 

### 3.2. Mitophagic Gene Signature

In cancer metabolism, mitophagy not only enables the removal of dysfunctional, depolarized, or damaged mitochondria, but also allows the reduction of mitochondrial biomass as an adaptive response to metabolic stress, such as hypoxia, starvation, and DNA damage that are present in the tumor. Besides providing all the essential nutrients to support tumor biology, through carbohydrate, protein, lipid, and nucleotide degradation [151], mitophagy is also involved in the control of ROS and calcium homeostasis for cell signaling and differentiation, and in the regulation of the balance between fermentative glycolysis and OXPHOS energy metabolic pathways [152,153].

In cancer patients, it has been observed that mitophagy processes are deregulated, but whether they behave as promoters of tumor formation or as suppressors seems to be highly dependent on the type of cancer and its metabolic context [151] (Table 6). In this regard, cancer stem cells, which are associated with the promotion of metastasis, therapy resistance, and tumor dormancy, make the most of mitophagy to sustain their self-renewal properties [154]. In lung or pancreatic cancer cells, the loss of the mitophagic proteins ATG7 or ATG5 significantly decreases the aggressiveness of the tumor due to the accumulation of defective mitochondria and an excessive accumulation of lipids, accounting for a greater alteration in the ß-oxidation of fatty acids [155]. 

Cancer cells are also characterized by the stabilization of the hypoxia-induced transcription factor HIF-1α, one of the main factors responsible for the change in energy metabolism from the oxidative metabolism to the aerobic glycolytic metabolism, also known as the Warburg effect [151]. Notably, HIF-1α also activates the transcriptional regulation of the mitophagy receptors NIX and BNIP3, which would decrease mitochondrial biomass, reducing the cells’ oxygen demand and thus promoting survival under hypoxic conditions. The HIF-1α signaling pathway is more active during the proliferation and metastasis of cancer cells, which is why high HIF-1α expression is associated with a poor clinical prognosis. Results found in U87 and Glio6 glioma cells cultured under hypoxia showed that 32 genes were upregulated, including the mitochondrial nuclear-encoded genes *BNIP3*, *NIX*, and *FAM162A* [174]. BNIP3 and NIX have been involved both in apoptosis and mitophagy [175,176,177,178,179,180,181], and FAM162A, only in apoptosis [182]. However, it has been mentioned as belonging to the same BH3-only family as NIX and BNIP3. Toustrup et al. [183] and Sorensen et al. [184] defined 15-gene hypoxia classifiers based on gene expression to predict prognosis and therapeutic response in various cancer types, because hypoxia is a common feature in solid tumors. They assessed four head and neck squamous cell carcinomas, three esophagus cancer cell lines, three prostate cancer cell lines, and six colon cancer cell lines, and *FAM162A, BNIP3,* and *NIX* genes were overexpressed [183,184]. To date, there is no information about those genes in thyroid cancer. Interestingly, FAM162A has been shown to be highly expressed in the SiHa uterine cervix cancer (UCC) cell line when HIF-1a is overexpressed. However, far from inducing apoptosis and cell death, the UCC SiHa cells increased their proliferation and migration capacities [185]. The expression of FAM162A was investigated in 85 human tissue samples of UCC and over 70% were positive for FAM162A by immunohistochemistry as well as for HIF-1a, suggesting that FAM162A participates in a molecular mechanism of hypoxia-regulated tumor development. In fact, FAM162A-positive tumors are associated with an advanced stage of cancer, parametrial invasion, and tumor recurrence, according to the International Federation of Gynecology and Obstetrics. In addition, the overall survival rate tended to be poorer in patients with FAM162A expression than in those without FAM162A expression [186].

## 4. Conclusions

Promising molecular markers for thyroid cancer associated with non-coding nuclear genes and mitochondria have been reviewed and proposed. From a diagnostic perspective, identifying novel molecular markers can enhance the accuracy of preoperative fine-needle aspiration samples. Among the increasing number of alternatives, and based on the number of studies performed with each RNA, miR-1, miR-2, miR-7, and miR-144, along with the long non-coding RNAs HOTAIR, MALAT1, and PTCSC3 emerge as the most robust and promising candidates. Regarding mitochondria, measurements and genetic analysis of mtDNA, as well as the expression of mitophagic proteins FAM162A, NIX, and BNIP3, will contribute to a better understanding of the biology and development of thyroid cancer and support current cancer diagnosis and prognosis, benefiting patients. It is envisioned that these novel biomarkers will complement the commonly used cancer risk/probability classification (Bethesda). Their results can then be compared with the definitive biopsy findings of patients operated on for thyroid cancer (Bethesda V and VI) and those operated on for suspected thyroid cancer (Bethesda III and IV). Thus, based on classifications of prognosis and recurrence, the novel molecular markers reviewed here can serve as indicators of aggressiveness in patients with papillary thyroid cancer. This can complement the guidelines from the National (Chilean Department of Health) and International (ATA and TNM) clinical guides for a more accurate assessment of the disease [187,188].

## Figures and Tables

**Table 1:** Definition of epidemiological and clinical terms used in this text.

Term	Definition
Incidence	Number of new cases of disease in a population during a period
Sensitivity	The probability that the test result will be positive when disease is present
Specificity	The probability that the test result will be negative when disease is absent
Positive Predictive Value (PPV)	The probability of having the disease with a positive test result
Negative Predictive Value (NPV)	Probability of not having the disease, with a negative test
Diagnostic Yield	Proportion of true positives cases within the study cohort, with a specific test

**Table 2:** Summary of the main characteristics of genetic classifiers.

Test	Type of Test	No. of Genes ^1^	Other Biomarkers	Clinical Application	Ref.
Afirma GSC/Afirma XA	RNA NGS (mRNA Expression)	1115 Genes	Mutation Hotspots + Fusions + Loss of heterozygosity	Afirma GSC is used to evaluate thyroid nodules with indeterminate cytology (Bethesda III/IV) to rule out benignity and thus help avoid unnecessary surgeries in benign cases. Therefore, it has diagnostic applications and can guide definitive treatment. Afirma XA is used for suspicious GSC samples and Bethesda V/VI.	[5,22,23]
Thyroseq V3	Targeted DNA and RNA NGS	112 Genes	>120 Fusions + 10 Copy Number Alterations + 19 Genes (Expression)	Distinguishes between malignant and benign lesions, helping to avoid unnecessary surgery during the diagnostic phase.	[24,25]
ThyGeNEXT/ThyraMIR	Targeted NGS + miRNA Expression	10 Genes	28 Fusions + 10 miRNAs (Expression)	To improve the risk stratification and diagnostic accuracy of indeterminate thyroid nodules. It detects mutations and micro RNAs associated with thyroid cancer and helps in selecting the right treatment. However, what sets it apart from other classifiers is its utilization for long-term monitoring. It is often combined with ThyraMIR, to enhance stratification and predictive value.	[5,26,27]
Thyroid Print	Quantitative Real-Time PCR (mRNA Expression)	10 Genes	N/A	Detects changes in gene expression to classify thyroid nodules’ malignancy. It provides additional information for diagnostic evaluation, which may influence treatment planning.	[28,29,30]

**^1^** Due to confidentiality policies, only the number of genes available in commercial classifiers is disclosed.

**Table 3:** miRNAs tested in commercial tests.

miRNA	ThyraMIR^®^	mir-THYpe^®^	Status in Thyroid Cancer
miR-21	+		Promoter of cancer. Higher in tumor [53,58]
miR-29	+		Suppressor of cancer. Reduced in tumor [53]
miR-31	+		Promoter of cancer. Higher in tumor [53,59]
miR-138	+		Suppressor of cancer. Reduced in tumor [53,60]
miR-139	+		Suppressor of cancer. Reduced in tumor [53]
miR-146	+	+	Promoter of cancer. Higher in tumor [53,56,59,61]
miR-152		+	Promoter of cancer. Higher in tumor [56,59]
miR-155	+	+	Suppressor of cancer. Reduced in tumor [53,56]
miR-181		+	Discriminator [56]
miR-200		+	Discriminator [56]
miR-204	+		Suppressor of cancer. Reduced in tumor [53]
miR-222	+		Promoter of cancer. Higher in tumor [53,61]
miR-375	+		Promoter of cancer. Higher in tumor [53,58]
miR-551	+		Promoter of cancer. Higher in tumor [53,59]

**Table 4:** Potential miRNAs biomarkers for thyroid cancer.

miRNA	Expression in Cancer	mRNA Target	Reference
miR-1	Downregulated	*CCND2*, *CXCR4* and *MET*	[63,64,65]
miR-7	Downregulated	*METTL7B*, *EGFR*	[60,66,67]
miR-2	Upregulated	*CBL* and beta-catenin	[68]
miR-30	Downregulated	*BCL9*, *SNAI1*, *SMAD2*, *TGFBR1* and *VIM*	[69,70]
miR-144	Downregulated	*TGFA*, *ZEB1*, *ZEB2*, *E2F8* and *WWTR1*	[71,72,73]
miR-198	Downregulated	*FGFR1*, *E2F8*	[74]
miR-485	Downregulated	*RAF1*	[75]
miR-488	Downregulated	*NUP205*	[76]
miR-613	Downregulated	*SPHK2*	[77]
miR-539	Downregulated	*MMP9*	[78]
miR-761	Downregulated	*PPME*	[79]
miR-873	Downregulated	*ZEB1*, *CXCL16*, *FSTL1*	[80,81,82]
miR-1183	Downregulated	*SOX2*	[83]

**Table 5:** Potential long non-coding RNA biomarkers for thyroid cancer.

lncRNA	Expression in Cancer	Function	Reference
*DUXAP8*	Upregulated	Increases proliferation, migration, and invasion.	[98,99]
*FAM230B*	Upregulated	Promotes migration and invasion in cancer cells through a mechanism involving the *Wnt* pathway.	[100]
*HOTAIR*	Upregulated	Controls early development- related genes. Increases growth, migration, and invasive.	[76,79,90,91]
*LINC00460*	Upregulated	Promotes cell proliferation, invasive properties, and epithelial-to-mesenchymal transition.	[75,77,78]
*MALAT1*	Upregulated	Increases cell proliferation, invasion, and migration.	[87,90,94,95]
*NEAT1*	Upregulated	Facilitates glycolytic metabolism, cell proliferation, and metastasis.	[90,101,102]
*MAPKAPK5-AS1*	Upregulated	Increases proliferation and invasion.	[103]
*OIP5-AS1*	Upregulated	Promotes cell proliferation, migration, and invasion.	[104]
*PTCSC3*	Downregulated	Tumor suppressor.	[88,97]
*XIST*	Upregulated	Inactivates X chromosome in female cells. Promotes proliferation and invasion.	[105,106]

**Table 6:** Mitophagic genes involved in cancer ^1^.

Mitophagic Protein	Function	Type of Cancer	Ref.
Pink/Parkin	Pro-tumorigenic	Breast, cervical, ovarian, lung, glioma, esophagus, melanoma	[156]
	Anti-tumorigenic	Liver, renal, pancreatic, endometrial	
FUNDC-1	Pro-tumorigenic	Cervical, hepatocarcinoma, breast, colorectal, leukemia, ovarian, pancreatic, Prostate, lung, and breast adenocarcinoma; and Glioblastoma	[157,158,159]
NIX	Pro-tumorigenic	Pancreatic	[160]
	Anti-tumorigenic	Osteosarcoma	[161]
BNIP3	Pro-tumorigenic	Lung, prostate, glioblastoma multiforme, cervical tumors, endometrial, breast carcinomas and gastric adenocarcinomas	[162,163,164]
	Anti-tumorigenic	Breast, Pancreatic	[165,166]
AMBRA	Pro-tumorigenic	Hepatocellular carcinoma, metastatic breast cancer, and medulloblastoma	[167]
	Anti-tumorigenic	Colorectal cancer cell, melanoma, and squamous cell carcinoma	[167]
FKBP8	Pro-tumorigenic	Hepatocellular Carcinoma	[168]
	Anti-tumorigenic	Schwannoma, melanoma	[169]
NLRX1	Pro-tumorigenic	Breast	[170]
	Anti-tumorigenic	Pancreatic	[171]
Prohibitin-2	Pro-tumorigenic	Lung, Cervical	[172,173]

^1^ This table presents only mitochondrial proteins involved in mitophagy, not proteins involved in the phagophore or autophagosome formation.

## Data Availability

Not applicable.
